# Peripheral intravenous catheter use in French emergency departments (CathIRU study): a multicentre cross-sectional study of non-indicated insertion rate and practice patterns

**DOI:** 10.1016/j.lanepe.2026.101674

**Published:** 2026-04-10

**Authors:** Bertrand Drugeon, Nicolas Marjanovic, Gabor Mihala, Sabrina Seguin, Guillaume Batiot, Victor Rouschmeyer, Camille Gerlier, Olivier Peyrony, Sophie Lefebvre, Jérémy Guenezan, Olivier Mimoz, CathIRU Group, Camille Raynaud, Maelle Morvan, Raphael Couvreur, Marine Demarquet, Johan Limousin, Fabien Rocard, Ludivine Carrouee, Vanessa Voisin, Claire Naulin-Leray, Fanny Granger, Clément Rochard, Ombeline Susong, Mathilde Chenu, Romain Adam, Matthieu Jourdain, Guillaume Ruiz, Xavier Dubucs, Charlotte Wemmert, Delphine Muller, Séverine Gosselin, Xavier Pereira, Céline Libe, Mathilde Papin, Camille Gerlier, Coraline Dujardin, Florent Noel, Eric Burggraff, Delphine Attia, Olivier Peyrony, Rym Hamed, Vincent Garrouste, Rébecca Mandin, Romain Futin, Hery Andrianjafy, Haithem Debbabi, Alexis Fremery, Pierre-yves Dupont, Frédéric Abbal, Angelo Giordano Kwakye Agyemang, Florian Negrello, Patrick Lesage, Mustapha Sebbane, Sylvain Thiriez, Christelle Galinski, Gwendoline Gonfrère, Delphine Levy, Maï Renault Fradin, Alice Bonnaure-Sorbier, Sewann Jeanne, Gwenola Rio, Said Laribi, Aurélien Leclercq-Martin, Lea Berment, Julie Samain, Bertrand Helios, Jessica Bortzmeyer, Mathieu Violeau, Nicolas Bounaud, Manon Kaced, Morgane Leon, Elise Prudhomme, Antonin Camphuis, Daniel Roux-Boniface, Virginie Restouilh, Arnaud Barth, Olivia Berrard-Lorrillere, Nicolas Cazes, Julien Yvon, William Hermann, Margaux Fertat, Isaure Fromont, Aline Henry, Isabelle Villard, Ayoub Touihar, Adrien Palomera, Pierre Van Caenegem, Max- Antoine Sartorius, Arthur Baisse, Deborah Jaeger, Pierre Goffin

**Affiliations:** a CHU de Poitiers, Service des Urgences Adultes – SAS 86 - Centre 15, Poitiers, France; b INSERM, U1070, Pharmacologie des Agents Anti-Infectieux et Resistance, Poitiers, France; c Alliance for Vascular Access Teaching and Research, Griffith University, Nathan, Australia; d Université de Poitiers, Faculté de Médecine et de Pharmacie, Poitiers, France; e INSERM CIC1402, CHU de Poitiers, Poitiers, France; f Centre for Health Services Research, Faculty of Health, Medicine & Behavioural Sciences, The University of Queensland, Woolloongabba, Australia; g Hôpital Paris-Saint Joseph, Service des Urgences, Paris, France; h Assistance Publique des Hôpitaux de Paris, Hôpital Saint Louis, Service des Urgences, Paris, France; i CHU de Montpellier, Service des Urgences, Montpellier, France

**Keywords:** Peripheral intravenous catheter, Emergency department, Unnecessary catheterisation, First-attempt insertion failure, A-DIVA score, Health care organisation

## Abstract

**Background:**

Peripheral intravenous catheters (PIVCs) are widely used in emergency departments (EDs), but many are inserted without subsequent use, exposing patients to avoidable risks and wasting resources. We determined the prevalence of non-indicated PIVCs in French EDs and identified associated patient-, treatment- and unit-specific factors.

**Methods:**

We conducted a multicentre, observational, cross-sectional (point prevalence) study across 81 EDs over 48 h in April 2025. Adult patients (age over than 18 years) having PIVC placement during their ED stay were eligible. Patients with an existing PIVC in place, life-threatening and conditions precluding data collection were excluded. For patients receiving multiple PIVCs, only the first successfully inserted PIVC was eligible. The primary outcome was the prevalence of non-indicated PIVCs, defined as (1) no use for fluids, intravenous medication, contrast agents, or blood transfusion administration within 24 h, or (2) absence of predefined high-risk deterioration scenarios. Multivariable regression identified the risk factors.

**Findings:**

Among 19,737 ED visits across 81 EDs, and after exclusion of 96 duplicate catheters and 403 catheters with missing primary endpoint, 3909 PIVCs inserted were analysed. Overall, 35% (1368/3910, 95% CI 30–36) were classified as non-indicated. Contributing factors included low-acuity triage and low nurse-to-patient ratios. Protective factors were age over 60 years, known difficult intravenous access (DIVA), high-acuity triage and dedicated vascular access teams.

**Interpretation:**

In this study, one-third of PIVCs were non-indicated and were mainly associated with low-acuity clinical presentations and organisational factors. These findings highlight substantial opportunities to improve the appropriateness of PIVC use in emergency care.

**Funding:**

10.13039/501100014811French Society of Emergency Medicine (€10,000 for administrative setup and data management).

**Research in context**

**Evidence before this study**

Before undertaking this study, we reviewed the available literature on peripheral intravenous catheter (PIVC) use in emergency departments. We searched PubMed from inception to December 2023 using combinations of terms related to peripheral intravenous catheter, emergency department, vascular access, unnecessary catheter, idle catheter, and failed insertion. Reference lists of relevant articles and narrative reviews were also screened. No language restrictions were applied.

The available evidence consisted mainly of single-centre observational studies, quality improvement initiatives, and a limited number of multicentre cohorts, primarily from Australia, Europe, and North America. These studies consistently reported that a substantial proportion (approximately 30–50%) of PIVCs placed in emergency settings were never used, but definitions of “unnecessary” varied and were often based on local criteria. Data on patient-level, operator-level, and organisational determinants of unnecessary PIVC placement were sparse and inconsistently reported. Few studies assessed centre-level variability, and none provided a large-scale, nationally representative snapshot of routine PIVC practices across emergency departments.

**Added value of this study**

This study provides the largest prospective, multicentre evaluation to date of PIVC practices in emergency departments, using a harmonised protocol across a large national research network. By applying predefined clinical criteria linked to actual catheter use within 24 h, it offers a pragmatic and reproducible estimate of the proportion of unnecessary PIVC placement in routine care.

Beyond prevalence, this study integrates patient, procedural, operator, and organisational factors within a single analytical framework, allowing identification of independent determinants of unnecessary catheterisation while accounting for centre-level clustering. It also quantifies inter-centre variability using intraclass correlation coefficients, highlighting the contribution of organisational context to practice variation.

**Implications of all the available evidence**

Taken together with existing literature, these findings confirm that unnecessary PIVC placement remains common in emergency care and is driven not only by clinical anticipation but also by organisational and system-level factors. The observed centre-level variability suggests that unnecessary catheterisation is, at least in part, a modifiable practice.

The combined evidence supports the implementation of structured decision-support approaches for PIVC insertion, and development of organisational enablers such as nurse-led anticipation protocols, catheter-related guidelines, and dedicated vascular access expertise. Future research should evaluate the effectiveness and sustainability of such interventions, assess their impact on patient-centred outcomes and infectious complications, and explore strategies to embed evidence-based vascular access decisions into everyday emergency department practice.

## Introduction

Peripheral intravenous catheters (PIVCs) are among the most widely used invasive medical devices worldwide, with over 2 billion units sold each year.^1^^,^^2^ In emergency departments (EDs), where clinical decisions often need to be made rapidly in uncertain clinical contexts, vascular access is routinely established in anticipation of potential diagnostic or therapeutic needs.^3^ Up to 70% of ED attendees receive a PIVC during their stay.^4^^,^^5^ While its insertion is considered a low-risk procedure, complications may occur in up to 40% of inserted devices.^6^ Complications contribute to increased patient discomfort, healthcare costs, and worse outcomes.^7^, ^8^, ^9^, ^10^

One of the most pressing and underappreciated issues in current PIVC practice is the extent of unnecessary catheterisation; i.e., catheters that are inserted but remain unused throughout their dwell time.^7^^,^^8^ Studies have found that 30–50% of PIVCs placed in EDs remain never used.^7^^,^^9^, ^10^, ^11^ However, most studies were single-centre or focused on targeted quality improvement interventions, raising doubts about the generalisability of these findings. Furthermore, associated factors with unnecessary catheter placement are generally poorly documented, limiting the implementation of preventive measures.

The CathIRU study was designed to address this gap by providing a snapshot of PIVC practices across French EDs affiliated with the Emergency Research Initiative (Initiative Recherche Urgences, IRU) research network of the French Emergency Medicine Society. Specifically, it aimed to estimate the proportion of unnecessary PIVC insertions based on predefined clinical criteria and actual usage within the first 24 h, and to identify their determinants. Throughout the manuscript, the term “non-indicated” is used in preference to “unnecessary” to reflect a neutral, indication-based assessment.

## Methods

### Study design

The IRU network comprises approximately 300 centres among the 691 French public or private EDs. Each year, a single research project is proposed to all members of IRU. Those wishing to participate nominate a local principal investigator (PI) responsible for implementation. To ensure consistency in data collection and study procedures with the present study, four videoconference training sessions were conducted with all PIs to clarify the protocol, review variable definitions, and address questions.

CathIRU was a multicentre, observational, cross-sectional (point-prevalence), study with a prespecified consecutive 48-h inclusion period commencing at 9 am on 3 April 2025. This design reflects the methodological approach of the research network, which favours short but intensive inclusion windows across numerous centres to rapidly generate representative epidemiological data. This study was designed and reported in accordance with the Strengthening the Reporting of Observational Studies in Epidemiology (STROBE) guidelines.^12^ The study protocol was registered with ClinicalTrials.gov (https://clinicaltrials.gov/study/NCT06851585). The study was approved by the Ethics and Scientific Committee on Health Data of Poitiers University Hospital in March 2025 (approval number 2025-03-01). The study was declared to the French data protection authority under reference methodology MR-004, as per French law. Given the observational nature of the study and the use of routinely collected data, the requirement for written informed consent was waived; patients were informed of the study and could object to the use of their data.

### Participants

All patients aged 18 years or older, requiring PIVC insertion in the ED, and not objecting to the collection of their data were enrolled. Patients with a prehospital vascular access device in place, with a life-threatening condition precluding data collection, or, to comply with French law, those without health insurance or under legal protection were excluded. For patients receiving multiple PIVCs during the ED visit, only the first successful catheter inserted was surveyed. PIVCs were inserted and managed in accordance with local protocols.

### Procedure

Data were prospectively collected from catheter insertion to 24 h post-insertion (or at ED discharge for outpatients) using an electronic Case Report Form (CRF) (REDCap, Yale University, New Haven, USA). Patient and treatment characteristics were selected based on literature review and clinical experience (Appendix 1). Investigators were instructed to enrol consecutive eligible patients during the 48-h study window; however, investigators were encouraged to prioritise consecutive enrolment whenever feasible.

### Outcomes

The primary outcome was PIVC indication status. PIVCs were classified as indicated when (a) used in the past 24 h for the administration of fluid, medication, contrast agent, or blood product, or (b) placed in patients meeting pre-defined criteria for potential clinical deterioration (respiratory/haemodynamic/neurological failure occurring within 30 min of triage, chest pain with abnormal electrocardiogram, postictal recovery following seizure, syncope, estimated blood loss >500 mL, overt active bleeding, high haemorrhagic risk under anticoagulant therapy, known congenital/acquired coagulopathy, known oesophageal varices). All other PIVCs were classified as non-indicated.^8^^,^^13^ These pre-defined high-risk clinical scenarios were derived from published Delphi-based expert consensus studies among emergency clinicians and were applied retrospectively by trained investigators based on clinical documentation, to avoid influencing catheter insertion decisions. In case of uncertainty, classification defaulted to non-indicated status (Appendix 2).

Key secondary outcomes included (i) patterns of effective PIVC use (medication, fluids, contrast agents, transfusion); (ii) patient-, operator- and centre-level factors associated with non-indicated PIVC placement (see list in Appendix 1).

### Statistical analysis

Continuous variables are presented as mean ± standard deviation (SD) or median [interquartile range, IQR], and categorical variables as counts and percentages. The overall prevalence of non-indicated PIVCs was estimated using a mixed-effects logistic regression model with a random intercept for centre, with marginal population-level estimates and 95% CIs derived from predicted marginal means.

The analysis of factors associated with non-indicated PIVC placement was conducted within a fully prespecified multivariable framework.^14^ All candidate covariates were defined a priori on the basis of existing literature, clinical plausibility, and expert consensus, independently of the observed data (Appendix 3).

Before model fitting, covariates were examined to ensure appropriate functional form and adequate category sizes; sparse categories were merged where necessary. Potential collider variables, identified on causal grounds, were deliberately not adjusted for. Multicollinearity was assessed using variance inflation factors (VIFs) calculated from complete-case logistic regression models. For categorical variables with more than two levels, generalized VIFs (GVIFs) were computed and rescaled as GVIFˆ (1/(2.df)). A conservative threshold of 4 was applied. No variable exceeded this threshold; all rescaled GVIFs were below 3 (Appendix 4).

The primary analysis consisted of a fully adjusted mixed-effects logistic regression model, with centre modelled as a random intercept to account for clustering and to enhance generalisability beyond the participating centres. Adjusted odds ratios (aORs) with 95% CIs are reported. The modelling strategy was explicitly focused on estimation rather than hypothesis testing, and no statistical significance thresholds were used to guide model specification.

Missing data were described for each covariate and across centres. Patients with missing data for the primary outcome were excluded from the primary analysis; baseline patient and centre characteristics of excluded versus included cases were compared to assess potential selection bias. The proportion of patients excluded from the complete-case multivariable analysis due to missing covariate data was quantified. Because the distribution of missingness varied between centres, multiple imputation by chained equations was performed under a missing-at-random (MAR) assumption, including all covariates, the outcome, and centre in the imputation model.^15^ Twenty imputed datasets were generated, and estimates were pooled using Rubin's rules.

Several prespecified sensitivity analyses were conducted to assess the robustness of the findings. First, centre was alternatively modelled as fixed effects while retaining the same covariate structure, and consistency in the direction and magnitude of effect estimates across specifications was used to support robustness. Second, to explore the impact of departures from the missing-at-random (MAR) assumption, best–worst and worst–best case analyses were performed for selected covariates for which a missing-not-at-random (MNAR) mechanism was clinically plausible, and estimates were compared with those from the primary analysis.^16^^,^^17^ Third, to address potential outcome misclassification related to anticipatory insertions, the outcome was redefined using a strict definition of necessity restricted to catheters actually used within 24 h of insertion, and multivariable analyses were repeated accordingly. In addition, predefined high-risk clinical scenarios in which anticipatory insertion may be clinically justified were excluded in a complementary analysis to assess the robustness of associations within a more homogeneous population.

We examined the typology of effective PIVC use, categorising catheters by their indication (e.g., medication, fluids, contrast agents, transfusion) and by whether multiple uses occurred.

All analyses were performed using R software (version 4.2.1 [2022-06-23], R Foundation for Statistical Computing, Vienna, Austria).

### Role of the funding source

This study received funding from the French Society of Emergency Medicine (SFMU) in the amount of €10,000, allocated to cover administrative set-up costs and data management activities. The funder had no role in the study design, data collection, data analysis, data interpretation, or writing of the report.

## Results

**Fig. 1:**
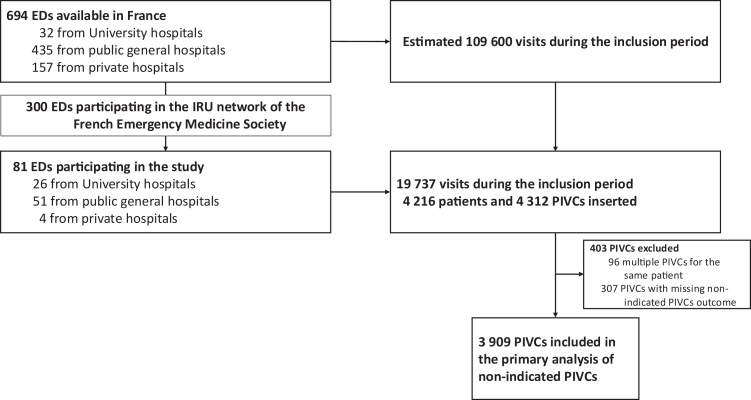
Flow chart. ED, Emergency Department; PIVC, Peripheral Intravenous Catheter; IRU, Initiative Recherche Urgences (Emergency Research Initiative). The flowchart focuses on adult patients with PIVC placement who met eligibility criteria and does not represent the overall proportion of ED patients receiving a PIVC.

A total of 81 EDs agreed to participate (Fig. 1). During the two-day inclusion period, 19,737 patients visited the participating EDs. Among them, 4216 adult patients with a newly inserted PIVC meeting the study eligibility criteria were enrolled, corresponding to 21% of all ED visits. This proportion reflects study enrolment relative to total ED activity during the inclusion window and should not be interpreted as the overall rate of PIVC insertion among ED attendees.

Participating centres were mostly general hospitals, with a wide variation in annual ED volumes and staffing levels (Table 1). Despite heterogeneity, most sites reported having local protocols for PIVC insertion and maintenance, while dedicated vascular access teams remained uncommon. Nurse-to-patient and prescriber-to-nurse ratios varied across institutions, particularly between day and night shifts.

**Table 1:** Hospital and ED characteristics.

	Overall n = 81 EDs
Hospital characteristics	
Number of hospital beds	852 ± 710
Hospital status	
University hospital	26 (32)
General public hospital	51 (63)
Private hospital	4 (5)
Existence of a catheter insertion protocol	55 (68)
Existence of a catheter maintenance protocol	55 (68)
Dedicated vascular access team	13 (16)
ED characteristics and staffing levels	
Annual number of ED visits	42,000 [30,000–53,277]
<15,000	6 (7)
15,000–50,000	46 (57)
>50,000	29 (36)
ED visits during the inclusion period	244 ± 131
Rate of nurses trained in ultrasound-guided insertion	5 (9)
Nurse-led vascular access anticipation protocol	10 (12)
Systematic catheter placement upon arrival	61 (75)
Nurse-to-patient daily ratio^a^	
Low	27 (33)
Moderate	28 (35)
High	26 (32)
Prescriber staffing and ratios	
Number of prescribers on day shift	9 ± 4
Number of prescribers on night shift	5 ± 3
Prescriber-to-nurse ratio (day shift)^b^	
Low	36 (44)
Moderate	18 (22)
High	27 (33)
Prescriber-to-nurse ratio (night shift)^c^	
Low	31 (39)
Moderate	33 (41)
High	16 (20)

Data are n (%) or Mean ± SD or Median [IQR]. ED denotes Emergency Department. Only one value was missing for the prescriber-to-nurse ratio during the night shift.

a Nurse-to-patient daily ratio categories were defined as follows: High = >1 nurse per 18 patients per day; Moderate = 1 nurse per 14–18 patients per day; Low = <1 nurse per 14 patients per day.

b Prescriber-to-nurse ratio (day shift) categories were defined as follows: Low = < 1 prescriber per nurse; Moderate = 1.0–1.33 prescribers per nurse; High = > 1.33 prescribers per nurse.

c Prescriber-to-nurse ratio (night shift) categories were defined as follows: Low = < 0.75 prescriber per nurse; Moderate = 0.75–1.00 prescriber per nurse; High = > 1.00 prescriber per nurse.

Included patients were 61 years on average, with an approximately even sex distribution and a high prevalence of overweight/obesity. Only a minority had specific conditions or treatments such as chemotherapy, dialysis, or drug abuse. Nearly half of the patients required hospital admission during the inclusion period. Most catheters were inserted during day shifts (72%) using the landmark technique, and the majority were 18–20G short catheters (97%) placed in the upper limb. Immediate complications (mostly minor haematoma or extravasation) were uncommon. Catheters were mainly (61%) inserted by nurses with ≥5 years of experience (Table 2, Appendix 5).

**Table 2:** Characteristics of patients, catheters and inserters among all included patients with PIVC.

	Overall N = 4216
Patients	
Male sex	1988/4212 (47)
Age, yrs	61 ± 25
<60	2148/4214 (51)
60–75	911/4214 (22)
>75	1155/4214 (27)
Body mass index, kg/m^2^^a^	26.4 ± 9.7
Weight insufficiency	204/3811 (5)
Normal weight	1667/3811 (44)
Overweight	1153/3811 (30)
Obesity	787/3811 (21)
Non cooperant patient^b^	148/4150 (4)
Drug abuse	80/4184 (2)
Chemotherapy	257/4175 (6)
Haemodialysis	39/4186 (1)
DIVA known	557/4075 (14)
Disposition	
Hospital discharge	2258/4170 (54)
Hospital admission	1840/4170 (45)
Discharge against medical advice	34/4170 (1)
Other (hospital transfer, death…)	38/4170 (1)
Catheter insertion	
Use of technology	16/4212 (<1)
Infrared device	0/16 (0)
Ultrasound	16/16 (100)
A-DIVA risk	
Low	2694/3139 (86)
Intermediate	368/3139 (12)
High	77/3139 (3)
Failure at first attempt	683/4183 (16)
Catheter types	
Insertion site	
Arm	180/4152 (4)
Cubital fossa	1436/4152 (35)
Forearm	1401/4152 (34)
Wrist	294/4152 (7)
Hand	831/4152 (20)
Other (jugular, lower limb)	10/4152 (<1)
Prior catheter unfunctional or incidentally withdrawn	90/4070 (2)
Inserters	
First operator's status	
Student	369/4214 (9)
Nurse	3831/4214 (91)
Resident	8/4214 (<1)
Junior doctor	2/4214 (<1)
Senior doctor	4/4214 (<1)

Data are n/N (%) or Mean ± SD.

a Body mass index categories were defined as follow: underweight (<20), normal weight (20–25), overweight (25.1–30), obesity (>30).

b Non-cooperative was defined as limited cooperation with the PIVC insertion procedure itself (e.g., agitation, inability to remain still), despite acceptance of data collection for the study, as assessed by the inserting clinician during routine clinical care.

After exclusion of 96 multiple catheters and 403 catheters with missing primary endpoint, 3909 PIVCs inserted in 3909 patients were included in final analyses. Patients excluded for missing primary outcome data had baseline characteristics broadly similar to those included (Appendix 6). Overall, 33% of patients had at least one missing covariate included in the multivariable model, with variation across centres.

In total, 1368 PIVCs (35%; 95% CI, 30–36%) were deemed non-indicated. Main appropriate indications were administration of treatment not available orally or in fasting patients and only 200 PIVCs were classified as indicated solely based on predefined high-risk clinical scenarios, corresponding to 5% of anticipated insertions (Table 3). Factors independently associated with non-indicated PIVC placement were low nurse-to-patient ratio and a non-urgent triage score. In contrast, patients aged over 60 years, history of difficult intravenous access (DIVA), involvement of a dedicated vascular access team, and high-acuity presentation at the ED were associated with reduced odds of non-indicated insertion (Table 4). Sensitivity analyses using the prespecified outcome definition were consistent with the primary findings for age, triage acuity, and involvement of a dedicated vascular access team, whereas the association with nurse-to-patient ratio showed some variability across model specifications. When the outcome definition was restricted to strict catheter use within 24 h, these associations were no longer observed (Appendix 7). Centre-level variability analyses showed wide heterogeneity in the prevalence of non-indicated PIVCs across participating EDs (Appendix 8). This heterogeneity was further quantified by an Intraclass Correlation Coefficient (ICC) of 0.12, indicating that approximately 12% of the total variance in non-indicated PIVC placement was attributable to differences between centres.

**Table 3:** Catheter utilisation.

Variables	Overall N = 4216
Use of the catheter during the first 24 h	
Intravenous fluid administration ≥ 1 L per 24 h	798/4081 (20)
Injection of contrast agent	981/4083 (24)
Non- substitutable per os administration or fasting patient	1479/4085 (37)
Blood products administration	96/4085 (2)
Catheter not used for treatment (unused or IV lock)^a^	1576/4084 (38)
Non-indicated catheter (primary outcome definition)	1368/3910 (35)
Clinically unstable condition^b^	200/3825 (5)
Respiratory, haemodynamic or neurologic failure within 30 min after triage	51/253 (20)
Chest pain with modified ECG	54/252 (21)
Post convulsive seizure recovery phase	20/253 (8)
Syncope	49/252 (19)
Active blood loss or over 500 mL lost	12/252 (5)
High haemorrhagic risk under anticoagulant treatment	21/251 (8)
Known coagulopathy and consultation related to haemorrhagic risk	12/252 (5)
Known oesophageal varices	0/252 (0)
Catheter removed following complication	47/4058 (1)

Data are n/N (%).

a Catheter non-use reflects the absence of actual administration through the device. Non-use does not necessarily imply that the catheter was non-indicated, as some catheters were placed in patients meeting predefined high-risk clinical criteria despite not being subsequently used.

b More than one unstable clinical condition could be reported for the same catheter.

**Table 4:** Multivariable analyses for factors associated with non-indicated catheter placement.

	Multivariable analysis
	aOR (95% CI), mixed-effects logistic regression (random intercept for centre)
Patient characteristics	
Age (yrs)	
<60	Reference
60–75	**0.83 (0.69–0.99)**
>75	**0.70 (0.59–0.83)**
Body mass index (kg/m^2^)	
Underweight	0.88 (0.61–1.25)
Normal weight	Reference
Overweight	0.90 (0.76–1.08)
Obesity	1.05 (0.86–1.28)
Non cooperative patient	0.92 (0.62–1.36)
Drug abuse	0.81 (0.47–1.40)
Chemotherapy	0.74 (0.54–1.01)
Haemodialysis	0.76 (0.35–1.69)
DIVA history	**0.80 (0.64–0.99)**
Insertion characteristics	
Triage score (3 levels)	
N1—High urgency	**0.60 (0.47–0.76)**
N2—Moderate urgency	Reference
N3—Low or non-urgent	**1.40 (1.16–1.70)**
Time of insertion	
Day shift	Reference
Night shift	1.00 (0.84–1.17)
Inserter characteristics	
First operator's years of experience	
<5 years	1.03 (0.85–1.25)
5–10 years	Reference
>10 years	1.03 (0.85–1.25)
Centre characteristics	
Hospital status	
University hospital	Reference
Other types of hospital	1.01 (0.74–1.37)
Existence of a catheter insertion protocol	1.13 (0.54–2.38)
Existence of a catheter maintenance protocol	0.83 (0.40–1.74)
Dedicated vascular access team	**0.50 (0.34–0.74)**
Staffing and activity levels	
Annual number of ED visits	
<15,000	0.55 (0.29–1.01)
15,000–50,000	Reference
>50,000	1.17 (0.84–1.62)
Nurse-led vascular access anticipation protocol	0.85 (0.56–1.27)
Catheter placement upon ED arrival	1.00 (0.71–1.40)
Nurse-to-patient daily ratio	
Low	**1.44 (1.00–2.06)**
Moderate	Reference
High	1.16 (0.80–1.69)
Prescriber-to-nurse ratio (day shift)	
Low	1.33 (0.91–1.95)
Moderate	Reference
High	1.10 (0.76–1.59)
Prescriber-to-nurse ratio (night shift)	
Low	0.82 (0.57–1.17)
Moderate	Reference
High	0.82 (0.56–1.21)

Adjusted odds ratios (aORs) and 95% confidence intervals (CIs) were estimated using a fully prespecified multivariable mixed-effects logistic regression model, with centre included as a random intercept to account for clustering. The primary analysis was conducted on multiply imputed datasets under a missing-at-random (MI-MAR) assumption (m = 20), with estimates pooled using Rubin's rules. No univariable screening, p-value–based selection, or stepwise procedures were used. Bold values are presented for ease of reading and do not imply statistical significance.

aOR, adjusted odds ratio; CI, confidence interval; ED, Emergency Department.

## Discussion

We found that 35% of PIVC placements in French EDs were classified as non-indicated, suggesting a substantial number of avoidable insertions and mirroring findings from other healthcare systems.^9^^,^^18^ This has important implications for patient safety and resource use. Local complications-including phlebitis, infiltration, pain, and occlusion-occur in up to 40% of catheters, even when not used.^6^^,^^19^^,^^20^ In addition, catheter-related bloodstream infections, though rare, increase 30-day mortality by approximately 20% and hospital costs by approximately €6500 (2025 values) per episode.^21^, ^22^, ^23^, ^24^ Finally, the annual cost of non-indicated PIVCs is considerable, estimated at over Au$300 million in Australian EDs, together with thousands of clinician hours spent on procedures that provided no therapeutic benefit.^10^

PIVC placement is frequently driven by habitual or anticipatory reasoning rather than immediate clinical need.^25^ This behavioural tendency is corroborated by prospective observational data from a French ED, where 24% of non-indicated PIVCs were inserted just in case.^11^ Our study adds to the existing body of evidence by quantifying the discrepancy between anticipated and actual need for intravenous access. Through multivariable modelling, we identified non-urgent or slightly urgent triage and absence of clinical complexity markers such as known DIVA or advanced age as predictors of non-indicated PIVC placement. Similar patterns have been reported elsewhere, with 74% non-indicated PIVCs among ambulatory patients and 27% among inpatients, and in qualitative studies describing “just-in-case” cannulation and blood-sampling only use.^3^^,^^11^ Although our dataset did not capture “blood-sampling only use” as an indication, the convergence of evidence points to practice norms rather than patient-specific need.

In parallel, organisational constraints appeared to be associated with non-indicated PIVC placement. Centres with lower nurse-to-patient ratios had higher rates of non-indicated PIVC placement, suggesting that task-oriented care under resource constraints may promote anticipatory catheter insertion as a default strategy to preserve patient flow, with limited subsequent re-evaluation once the device is in place.^3^ However, given the cross-sectional design, these associations should be interpreted cautiously, as they may also be influenced by unmeasured confounding factors such as crowding, local workflows, or differences in patient case-mix. Together, these results highlight the need for a dual approach: reinforcing individual clinical reasoning and establishing system-level enablers to discourage non-indicated catheter placement in low-risk patients.

When the outcome definition was restricted to strict catheter use within 24 h, associations with clinical and organisational factors were no longer observed. This discrepancy may reflected differences in the classification of anticipatory catheter placements. In the primary definition derived from the Delphi consensus, catheters inserted in predefined high-risk clinical deterioration scenarios were considered clinically justified even if ultimately unused. By contrast, the stricter definition classifies these insertions as non-indicated solely because they were not use. Although such scenarios represented only a small proportion of cases (approximately 5% of patients with a PIVC), their inclusion may have introduced clinically justified insertions into the non-indicated category, thereby diluting associations with clinical and organisational variables. In addition, organisational factors such as triage acuity, nurse-to-patient ratios, or the presence of specialised vascular access teams are more likely to influence the decision to insert a catheter rather than the subsequent clinical need for intravenous therapy, which is primarily driven by patient-level factors and the clinical course after ED assessment. Together, these findings suggest that organisational context may primarily influence anticipatory catheter placement rather than strictly unnecessary catheter use, underscoring the importance of clinically grounded definitions when evaluating the appropriateness of PIVC insertion in emergency settings.

In response to growing concerns regarding the overuse of PIVCs in EDs, several institutions have developed structured decision aids to support more judicious catheter placement. One commonly cited framework recommends inserting PIVC only when the clinician is at least 80% confident it will be used within 24 h for intravenous medication, fluids, blood products or contrast administration.^8^ Educational interventions based on this principle have been shown to improve practice patterns: in one study, implementation of a multimodal program incorporating this rule led to a 25% reduction in overall insertion rates and higher proportion of appropriately used catheters.^7^ Yet, despite these promising results, the benefits of such strategies vary from one institution to another and are limited in time. Several initiatives have focused on raising awareness among clinicians regarding the risks associated with PIVCs over-prescription, including patient discomfort, infection risk, and resource waste. However, these awareness campaigns tend to be sporadic and rarely embedded into routine clinical workflows. Yet even these structured approaches often face significant organisational barriers, including competing priorities, staff turnover, and variability in local leadership engagement. Furthermore, few studies to date have assessed the long-term clinical consequences of non-indicated PIVC placement, particularly with regard to infectious complications.^26^ These findings call for targeted efforts to harmonise insertion criteria and support evidence-based vascular access decisions across emergency settings.

Several limitations should be acknowledged. Firstly, the cross-sectional design limits causal inference and provides only a two-day snapshot of PIVC practices, which may not capture longer-term or seasonal variations. Although the “flash” design promoted wide participation, it may have introduced selection bias, as inclusion depended on local team capacity. Secondly, in high-throughput centres, not all eligible insertions could be captured and the total number of eligible PIVCs was not recorded, preventing estimation of the inclusion proportion and potentially introducing selection bias. Nevertheless, more than one in five French ED patients were enrolled, with variability between centres suggesting differences in case-mix and local practices (Appendix 8). Although this proportion appears lower than in some international cohorts,^5^ several factors explain this difference: paediatric patients accounting for approximately one quarter of ED visits^27^ were excluded, some adults already had vascular access devices, and others were not eligible or declined participation. Moreover, most ED visits in France do not require intravenous therapy, supporting an indication-based rather than anticipatory approach to PIVC placement. Within this context, the included population likely approximates patients for whom new PIVC insertion could reasonably be expected. Thirdly, only the first catheter per patient was analysed to preserve independence of observations, which may have underestimated repeated cannulation or cumulative complications, although subsequent insertions in the ED generally reflect clear clinical indications. Fourthly, information bias is another concern, as variables such as A-DIVA components, operator experience, and catheter use relied on self-report or documentation. Fifthly, the operational definition of non-indicated PIVCs may have resulted in limited misclassification, as the appropriateness of intravenous therapy depends on patient-specific factors (e.g., fasting status, swallowing disorders, clinical tolerance) and local medication availability that could not be fully reconstructed retrospectively. Sixthly, follow-up was limited to 24 h, preventing assessment of longer-term complications, including mechanical or infectious events and patient-reported outcomes. Seventhly, the professional category of the inserter was not analysed as an independent factor due to limited variability, and staffing variables were collected at the centre level rather than at the time of insertion, which may have attenuated associations. Missing covariate data were handled using multiple imputation, with results consistent with complete-case analyses. Finally, extrapolation to other healthcare systems should remain cautious given differences in staffing, vascular access protocols, and training.

This study has important strengths. Firstly, to our knowledge, it is the largest multicentre investigation to date of PIVC practices in EDs, across diverse geographic and organisational contexts, strengthening the representativeness of our findings. Secondly, the use of an electronic CRF and pre-study investigator training promoted consistency in data collection. The simultaneous recording of patient-, operator-, and centre-level data provided a comprehensive view of the determinants of non-indicated catheter placement. In addition to variables commonly reported in the literature, this study deliberately incorporated novel, clinically-informed variables to explore under-investigated determinants of PIVC use. This approach not only strengthens the contextual relevance of the findings but also represents a methodological innovation that broadens the current understanding of vascular access practices in emergency settings.

### Conclusion

This large multicentre prospective study shows that one in three PIVCs inserted in French EDs is non-indicated, reflecting a persistent reliance on anticipatory catheter placement. Age, low-acuity presentations and suboptimal organisational factors were key drivers. Addressing this gap will require coordinated strategies combining decision-support tools and organisational changes to support more judicious vascular access practices in emergency care. Future national initiatives should integrate these findings to promote safer and cheaper, evidence-based vascular access decisions in emergency care.

## Contributors

BD, OM, GB, SS, VR, CG, OP, SL made substantial contributions to conception and design, or acquisition of data, or analysis and interpretation of data.

BD, OM, NM, JG, GM were involved in drafting the manuscript or revising it critically for important intellectual content.

BD, OM, NM, JG, SS, GB, VR, GM, CG, OP, SL gave final approval of the version to be published. Each author has participated sufficiently in the work to take public responsibility for appropriate portions of the content.

BD agreed to be accountable for all aspects of the work, ensuring that questions related to the accuracy or integrity of any part of the work are appropriately investigated and resolved.

## Data sharing statement

Deidentified individual participant data collected for this study, along with a data dictionary defining each variable, will be made available to others. Additional related documents, including the study protocol and the statistical analysis plan, will also be available.

Data will be available from the time of publication. Requests for data access should be addressed to the corresponding author. Data will be shared with qualified researchers for scientifically sound proposals related to the objectives of the study. Access will be granted after review and approval of a proposal and following the signing of a data access agreement. Data will be provided without investigator support.

Statistical analysis code used for the analyses will be made available upon reasonable request.

## Declaration of interests

The authors declare no competing interests.

## Appendix A Supplementary data

All Appendix

List of Collaborators
