# Supplementary material for: Peripheral intravenous catheter use in French emergency departments (CathIRU study): a multicentre cross-sectional study of non-indicated insertion rate and practice patterns
Source: Lancet Reg Health Eur. 2026 Apr 10;65:101674. doi: 10.1016/j.lanepe.2026.101674 (PMC13091371; doi:10.1016/j.lanepe.2026.101674)
Supplement: All Appendix [file mmc1.docx]

Table of content

[Appendix 1. Detailed description of all collected variables. 2](#_Toc223726210)

[Appendix 2. Algorithm for classification of PIVC indication 6](#_Toc223726211)

[Appendix 3. Covariates assessed and assessment of inter-centre variability. 7](#_Toc223726212)

[Appendix 4. Multicollinearity diagnostics. 8](#_Toc223726213)

[Appendix 5. Other characteristics of centres, patients, catheters and inserters. 9](#_Toc223726214)

[Appendix 6. Missing data patterns and handling. 12](#_Toc223726215)

[Appendix 7. Multivariable sensitivity analyses for factors associated with non-indicated catheter insertion. 16](#_Toc223726216)

[Appendix 8. Centre-level variability in non-indicated PIVC placement (Panel A) and enrolled patients (Panel B) 18](#_Toc223726217)

# **Appendix 1. Detailed description of all collected variables.**

**PATIENT-LEVEL DATA**

Patient-level data included patient, insertion, catheter and operator characteristics. Additional categorical variables were derived to refine the analyses.

**Patient characteristics**

Demographic data included age (categorised as <60, 60–75, and >75 years), body mass index (underweight [<20], normal weight [20-25], overweight [25,1-30], obesity [>30], based on WHO definitions) (1), body weight (<75 kg, 75–100 kg, >100 kg), and skin phototype, classified into three groups (pale, intermediate, dark). Clinical presentation was captured through the triage score assigned at ED arrival. To ensure comparability across sites, both the French Emergency Nurses Classification for Hospitalisation (FRENCH) and the Classification Infirmière des Malades aux Urgences (CIMU) scales were accepted. The FRENCH scale comprises five categories, from level 1 (life-threatening emergency) to level 5 (non-urgent), while CIMU contains five similar gradations. To harmonise these systems for analysis, we created a unified five-level classification and a simplified three-level categorisation: high urgency (levels 1–2, patients at risk of deterioration within minutes to 1 hour after admission), moderate urgency (level 3, risk of deterioration within 24 hours), and low/no urgency (levels 4–5, no expected deterioration) (2).

**Insertion characteristics**

Insertion-related variables included the time of catheter placement, categorised into two periods: “day” (08:00 AM to 07:59 PM) and “night” (08:00 PM to 07:59 AM), and the urgency level of the clinical situation, based on harmonisation of triage scores across centres using either the FRENCH or CIMU scale. This harmonisation enabled the creation of both a five-level classification (from “N1 – life-threatening emergency” to “N5 – non-urgent”) and a three-level simplified version (high, moderate, low/no urgency). The modified Adult-Difficult IntraVascular Access (A-DIVA) score was employed to stratify patients based on their predicted risk of first-attempt failure in PIVC insertion. This clinical tool is derived from validated criteria and integrates five binary items: (1) history of difficult vascular access (DIVA), (2) anticipation of failure by the healthcare provider, (3) absence of a palpable vein on the upper limb after tourniquet application, (4) absence of a visible vein under the same conditions, and (5) maximal dilated vein diameter <3 mm. Each criterion contributes one point to the total score (range: 0–5). Patients were classified into three predefined risk categories: Low risk (0–1 criteria); Moderate risk (2–3 criteria); High risk (4–5 criteria) (3,4). To minimise behavioural bias and avoid prompting the use of adjunctive techniques such as ultrasound, the components of the score were not presented as a unified scale in the case report form (CRF) but were instead dissociated and embedded within broader clinical documentation. The score was reconstructed post hoc during the analysis phase. The presence of cannulation on the dominant limb was captured using a binary variable. First-attempt insertion failure was defined as the inability to obtain a functional peripheral intravenous catheter, i.e., one allowing immediate and reliable fluid injection, during the initial skin puncture, requiring one or more additional punctures to achieve successful vascular access (5). Overall failure rate was defined as complete failure of catheter insertion after all attempts, resulting in abandonment of the procedure.

**Catheter characteristics**

Catheter-related variables included catheter type, gauge, length, insertion site, and whether it was connected to an infusion line at the time of data collection. The catheter system was categorised as open, closed, or integrated. Open systems refer to catheters connected to a stopcock (or three-way tap), which require manual assembly and are more prone to manipulation and contamination. Closed systems are equipped with a valve at the catheter hub, with or without extension tubing, allowing needleless access and reducing the risk of disconnection and exposure to pathogens (6). Integrated systems comprise a pre-assembled unit including the catheter, extension tubing, and a valve, manufactured as a single sterile device (7). This design minimises the need for post-insertion assembly, thereby reducing manipulation and the associated risk of contamination (8). Gauge size was recorded and classified into standard categories (14G, 16G, 18G, 20G, 22G, 24G). Length was dichotomised into short (≤6 cm) and long (>6 cm) catheters (9). The anatomical site of insertion was documented and grouped as follows: hand, wrist, forearm, cubital fossa, arm, and other (e.g., jugular or lower limb) (10). Whether the catheter was placed following the dysfunction or accidental withdrawal of a previous device was also recorded. Finally, use of adjunctive technologies (infrared or ultrasound) and connection to an infusion system were captured as binary variables.

**Operator characteristics**

Operator-related variables included the operator’s professional category (nurse vs other), their professional experience (defined as the number of years in a role allowing independent catheter insertion), and the cumulative number of catheter insertions performed during their career. Although operator profession was recorded descriptively, it was not included in regression analyses because the vast majority of PIVCs were inserted by nurses, resulting in insufficient variability for meaningful modelling; operator experience was therefore used as the primary operator-level variable. Professional experience was categorised into three groups: <5 years, 5–10 years, and >10 years. Cumulative procedural volume was similarly classified into <100, 100–800, and >800 lifetime catheter insertions. These variables were chosen to reflect both formal experience and practical procedural exposure, two distinct dimensions likely to influence insertion success.

**Clinical indications**

Data collected through the case report form captured both patient disposition and detailed information on PIVC utilisation within the first 24 hours following insertion (or until ED discharge for outpatients). Patient disposition at the end of the ED stay was recorded as discharge home, hospital admission, discharge against medical advice.

PIVC use was categorised according to predefined clinical indications. Effective utilisation included administration of intravenous fluids or volume expansion (≥1 L within 24 hours), injection of contrast agents for diagnostic imaging, administration of intravenous medications not substitutable by oral therapy or in fasting patients, and administration of blood products. Catheters were also classified as unused when none of these indications occurred, including situations described as “keep vein open”, prophylactic placement, or removal because the catheter was deemed non-indicated. Early catheter removal due to complications (e.g., dislodgement, dysfunction, or pain) was specifically recorded.

For catheters that were not used within 24 hours, the presence of predefined high-risk clinical situations justifying anticipatory vascular access was systematically assessed. These included acute respiratory, haemodynamic, or neurological failure occurring within 30 minutes of triage; chest pain associated with electrocardiographic abnormalities; postictal recovery following seizure; syncope; estimated blood loss greater than 500 mL or active bleeding; high haemorrhagic risk under anticoagulant therapy; known congenital or acquired coagulopathy associated with bleeding risk; and known oesophageal varices. These criteria were selected based on prior literature and expert consensus and were used to distinguish potentially justified anticipatory catheterisation from non-indicated PIVC placement.

**CENTRE-LEVEL DATA**

Centre-level data covered organisational features such as staffing levels, availability of ultrasound equipment, local protocol for vascular access, and presence of a dedicated vascular access team.

Several derived variables were created to describe structural and organisational characteristics of participating EDs. The average number of ED visits per day was calculated by dividing the annual number of visits by 365 (see table 1 and 2 for distribution of nurse-to-patient ratio across centres)). A nurse-to-patient ratio was computed by dividing the number of nurses per day by the mean daily number of ED visits, calculated from the annual ED attendance. This ratio was then categorised into tertiles based on its empirical distribution across centres (low, moderate, high). Similarly, the number of medical prescribers (residents and physicians) was summed separately for the day and night shifts. The prescriber-to-nurse ratios were calculated for both periods and categorised into tertiles using the same approach. EDs were also classified according to their annual patient volume into three predefined categories: <15 000, 15 000–50 000, and >50 000 visits per year (11,12). Systematic catheter placement upon arrival was recorded as a centre-level organisational practice and analysed accordingly. Lastly, the rate of intravenous catheter placement during the study inclusion period was calculated as the proportion of enrolled patients (each corresponding to a catheter insertion) relative to the total number of ED visits over the same period, expressed as a percentage.

**Table 1. Continuous distribution of nurse-to-patient ratios (centre-level)**

| **Number of centres** | **Median ratio** | **Q1** | **Q3** | **Minimum** | **Maximum** |
| --- | --- | --- | --- | --- | --- |
| 81 | 0·064 | 0·054 | 0·074 | 0·006 | 0·183 |

**Table 2. Distribution by terciles of nurse-to-patient ratios (centre-level categorisation)**

| **Nurse-to-patient ratio category** | **Centres (n)** | **% of centres** |
| --- | --- | --- |
| Low | 26 | 32·1 |
| Moderate | 26 | 32·1 |
| High | 29 | 35·8 |
| **Total** | **81** | **100** |

**REFERENCES**

1. Obesity and overweight [Internet]. [cited 2025 Aug 5]. Available from: https://www.who.int/news-room/fact-sheets/detail/obesity-and-overweight

2. Taboulet P, Maillard-Acker C, Ranchon G, Goddet S, Dufau R, Vincent-Cassy C, et al. Triage des patients à l’accueil d’une structure d’urgences. Présentation de l’échelle de tri élaborée par la Société française de médecine d’urgence : la FRench Emergency Nurses Classification in Hospital (FRENCH). Ann Fr Med Urgence. 2019 Jan;9(1):51–9.

3. van Loon FHJ, Puijn LAPM, Houterman S, Bouwman ARA. Development of the A-DIVA Scale: A Clinical Predictive Scale to Identify Difficult Intravenous Access in Adult Patients Based on Clinical Observations. Medicine. 2016 Apr;95(16):e3428.

4. van Loon F, van Hooff L, de Boer H, Koopman S, Buise M, Korsten H, et al. The Modified A-DIVA Scale as a Predictive Tool for Prospective Identification of Adult Patients at Risk of a Difficult Intravenous Access: A Multicenter Validation Study. JCM. 2019 Jan 26;8(2):144.

5. Zhang Z, Wang X, Zhang L, Lou X, Su X, Wang X, et al. Infrared Vein Imaging for Insertion of Peripheral Intravenous Catheter for Patients Requiring Isolation for Severe Acute Respiratory Syndrome Coronavirus 2 Infection: A Nonrandomized Clinical Trial. Journal of Emergency Nursing. 2022 Mar;48(2):159–66.

6. González López JL, Arribi Vilela A, Fernández Del Palacio E, Olivares Corral J, Benedicto Martí C, Herrera Portal P. Indwell times, complications and costs of open vs closed safety peripheral intravenous catheters: a randomized study. Journal of Hospital Infection. 2014 Feb;86(2):117–26.

7. Gidaro A, Quici M, Giustivi D, Pinelli F, Samartin F, Casella F, et al. Integrated short peripheral intravenous cannulas and risk of catheter failure: A systematic review and meta-analysis. J Vasc Access. 2024 Jan 2;11297298231218468.

8. Guenezan J, Marjanovic N, Drugeon B, Neill RO, Liuu E, Roblot F, et al. Chlorhexidine plus alcohol versus povidone iodine plus alcohol, combined or not with innovative devices, for prevention of short-term peripheral venous catheter infection and failure (CLEAN 3 study): an investigator-initiated, open-label, single centre, randomised-controlled, two-by-two factorial trial. Lancet Infect Dis. 2021;21(7):1038–48.

9. Zingg W, Barton A, Bitmead J, Eggimann P, Pujol M, Simon A, et al. Best practice in the use of peripheral venous catheters: A scoping review and expert consensus. Infection Prevention in Practice. 2023 Jun;5(2):100271.

10. Drugeon B, Marjanovic N, Boisson M, Buetti N, Mimoz O, Guenezan J. Insertion site and risk of peripheral intravenous catheter colonization and/or local infection: a post hoc analysis of the CLEAN 3 study including more than 800 catheters. Antimicrob Resist Infect Control. 2024 Jun 5;13(1):57.

11. Members of the Public Health and Injury Prevention Committee. Public Health Impact of ED Crowding and Boarding of Inpatients. American College of Emergency Physicians; 2009.

12. Metten MA, Lelièvre F, Galland C, Buyck JF, San Miguel M. Panorama Urgences 2023 - Activité des services d’urgences des Pays de la Loire. Observatoire régional des urgences des Pays de la Loire; 2024.

# **Appendix 2. Algorithm for classification of PIVC indication**

# **Appendix 3. Covariates assessed and assessment of inter-centre variability.**

**Covariates assessed**

The following covariates were prespecified a priori and included in the multivariable mixed-effects logistic regression models based on clinical relevance, existing literature, and expert consensus: age category; body mass index category; patient cooperation; drug abuse; ongoing chemotherapy; haemodialysis; history of difficult intravenous access (DIVA); triage acuity (three-level classification); time of insertion (day vs night); inserter experience (<5, 5–10, >10 years); hospital status (university vs other); existence of catheter insertion and maintenance protocols; presence of a dedicated vascular access team; annual emergency department visit volume (<15,000; 15,000–50,000; >50,000); nurse-led vascular access anticipation protocol; catheter placement upon arrival; nurse-to-patient ratio; and prescriber-to-nurse ratios during day and night shifts. No univariable screening, p-value–based filtering, or stepwise selection procedures were used.

**Assessment of inter-centre variability**

To assess inter-centre variability, a complementary descriptive analysis was performed by plotting the proportion of non-indicated PIVCs for each participating emergency department, with corresponding 95% confidence intervals. In addition, inter-centre variability was quantified by estimating intraclass correlation coefficients (ICCs) for non-indicated PIVC placement using mixed-effects logistic regression null models including a random intercept for centre. For this binary outcome, ICCs were derived from the centre-level variance component assuming a logistic residual variance of π²/3 and interpreted as the proportion of total outcome variance attributable to between-centre differences.

# **Appendix 4. Multicollinearity diagnostics.**

Multicollinearity diagnostics were performed for the prespecified multivariable mixed-effects logistic regression model. Because all covariates were categorical, generalized variance inflation factors (GVIFs) were calculated from the corresponding complete-case logistic regression model prior to multiple imputation. For variables with more than two levels (Df > 1), GVIFs were rescaled as GVIF^(1/(2·Df)). For binary variables (Df = 1), the adjusted GVIF corresponds to √GVIF. The adjusted GVIF was used for interpretation in all cases.

All adjusted GVIF values remained well below the prespecified conservative threshold of 4. The highest adjusted GVIF observed was 2·95 (catheter maintenance protocol), indicating no concerning multicollinearity among covariates. No exact aliasing was detected. These findings suggest that multicollinearity is unlikely to have materially influenced the stability or interpretation of the multivariable model estimates.

**Table 1. Multicollinearity diagnostics.**

| **term** | **GVIF** | **Df** | **GVIF_adj** |
| --- | --- | --- | --- |
| **Age (yrs)** | 1·076 | 2 | 1·018 |
| **Body mass index (Kg/m2)** | 1·056 | 3 | 1·009 |
| **Non cooperative patient** | 1·008 | 1 | 1·004 |
| **Drug abuse** | 1·028 | 1 | 1·014 |
| **Chemotherapy** | 1·033 | 1 | 1·016 |
| **Haemodialysis** | 1·022 | 1 | 1·011 |
| **DIVA history** | 1·045 | 1 | 1·022 |
| **Triage score (3 levels)** | 1·076 | 2 | 1·019 |
| **Time of insertion** | 1·027 | 1 | 1·013 |
| **First operator's years of experience** | 1·076 | 2 | 1·018 |
| **Hospital status** | 1·525 | 1 | 1·235 |
| **Existence of a catheter insertion protocol** | 8·265 | 1 | 2·875 |
| **Existence of a catheter maintenance protocol** | 8·697 | 1 | 2·949 |
| **Dedicated vascular access team** | 1·607 | 1 | 1·268 |
| **Annual number of ED visits** | 1·811 | 2 | 1·160 |
| **Nurse-led vascular access anticipation protocol** | 1·216 | 1 | 1·102 |
| **Catheter placement upon ED arrival** | 1·370 | 1 | 1·170 |
| **Nurse-to-patient daily ratio** | 2·406 | 2 | 1·245 |
| **Prescriber-to-nurse ratio (day shift)** | 2·444 | 2 | 1·250 |
| **Prescriber-to-nurse ratio (night shift)** | 2·937 | 2 | 1·309 |

# **Appendix 5. Other characteristics of centres, patients, catheters and inserters.**

|  | | | | **Overall** |
| --- | --- | --- | --- | --- |
|  |  |  |  | **N = 81 centres / 4 216 patients** |
| **CENTRES** | | | | |
| **Total number of nurses** | | | | 47 ± 25 |
| Number of nurses on day shift | | | | 8 ± 4 |
| Number of nurses on night shift | | | | 6 ± 4 |
| **Total number of residents** | | | | 9 ± 6 |
| Number of residents on day shift | | | | 4 ± 2 |
| Number of residents on night shift | | | | 2 ± 2 |
| **Number of junior physicians** | | | | 3 ± 4 |
| **Total number of senior physicians** | | | | 30 ± 19 |
| Number of physicians on day shift | | | | 5 ± 3 |
| Number of physicians on night shift | | | | 3 ± 2 |
| **PATIENTS** | | | | |
| **Weigt, kg** | | |  | 73 ± 19 |
|  | *<75* | | | 2 171/3 914 (55) |
|  | *75-100* | | | 1 485/3 914 (38) |
|  | *>100* | | | 258/3 914 (7) |
| **Size, cm** | | |  | 166 ± 18 |
| **Triage score ‡** | | |  |  |
| *CIMU scale* | | |  | 979/4 146 (24) |
|  | *CIMU 1* | | | 25/975 (3) |
|  | *CIMU 2* | | | 204/975 (21) |
|  | *CIMU 3* | | | 492/975 (50) |
|  | *CIMU 4* | | | 222/975 (23) |
|  | *CIMU 5* | | | 32/975 (3) |
| *French scale* | | |  | 2 764/4 179 (66) |
|  | *French 1* | | | 47/2 737 (2) |
|  | *French 2* | | | 482/2 737 (18) |
|  | *French 3A* | | | 709/2 737 (26) |
|  | *French 3B* | | | 1 227/2 737 (45) |
|  | *French 4* | | | 237/2 737 (9) |
|  | *French 5* | | | 35/2 737 (1) |
| *Other scale* | | |  | 444/4 162 (11) |
| **Dominant side** | | | |  |
|  | *Right* | | | 3 160/4 112 (75) |
|  | *Left* | | | 309/4 112 (8) |
|  | *Both* | | | 80/4 112 (2) |
|  | *Not known* | | | 563/4 112 (14) |
| **Skin colour** Fitzpatrick scale | | | | |
|  | | *Very pale* | | 270/3 036 (9) |
|  | | *Pale* | | 1 692/3 036 (56) |
|  | | *Tanned* | | 631/3 036 (21) |
|  | | *Olive* | | 221/3 036 (7) |
|  | | *Brown* | | 152/3 036 (5) |
|  | | *Dark brown* | | 70/3 036 (2) |
|  | | *Pale skin* | | 1 962/3 036 (65) |
|  | | *Middle skin* | | 852/3 036 (28) |
|  | | *Dark skin* | | 222/3 036 (7) |
| **CATHETER INSERTION** | | | | |
| **Total number of operators** | | | | 1.5 ± 0.7 |
| **Number of attemps** | | | | 1 ± 1 |
| **Time of insertion** | | | |  |
|  | | *Day shift* | | 3 004/4 162 (72) |
|  | | *Night shift* | | 1 158/4 162 (28) |
| **Insertion side** | | | |  |
|  | | *Right* | | 1 967/4 116 (48) |
|  | | *Left* | | 2 149/4 116 (52) |
| **A-DIVA score** | | | |  |
|  | | Score 0 | | 2 019/3 139 (64) |
|  | | Score 1 | | 675/3 139 (22) |
|  | | Score 2 | | 258/3 139 (8) |
|  | | Score 3 | | 110/3 139 (4) |
|  | | Score 4 | | 62/3 139 (2) |
|  | | Score 5 | | 15/3 139 (1) |
| **Dominant side canuled** | | | | 312/4 054 (8) |
| **Vascular access team required** | | | | 6/4 200 (<1) |
| **Immediate complication** | | | | 162/4 151 (4) |
|  | | *Paresthesia* | | 1/4216 (1) |
|  | | *Hematoma* | | 145/4 216 (90) |
|  | | *Arterial punction* | | 3/4 216 (2) |
|  | | *Extravasation* | | 20/4 216 (12) |
| **Total failure** | | | | 18/4 208 (<1) |
| **Use of alternative vascular access** | | | | 3/4 215 (<1) |
| **CATHETER TYPES** | | | | |
| **Catheter system** | | | |  |
|  | | *Closed* | | 2 106/4135 (51) |
|  | | *Open* | | 1 947/4 135 (47) |
|  | | *Integrated* | | 82/4 135 (2) |
| **Catheter connected to perfusion lign** | | | | 1 572/4 134 (38) |
| **Catheter's lenght** | | | |  |
|  | | *Short* | | 4 083/4 166 (98) |
|  | | *Long > 6cm* | | 83/4 166 (2) |
| **Catheter's size (gauge)** | | | | |
|  | | *14* | | 1/4 095 (<1) |
|  | | *16* | | 29/4 095 (1) |
|  | | *18* | | 1 770/4 095 (43) |
|  | | *20* | | 2 197/4 095 (54) |
|  | | *22* | | 96/4 095 (2) |
|  | | *24* | | 2/4 095 (<1) |
| **INSERTERS** | | | | |
| **First operator's age** | | | | 32 ± 9 |
| **First operator's sex (female)** | | | | 3 240/4 207 (77) |
| **First operator's years of experience** | | | | 9 ± 8 |
|  | | *<5 years* | | 1 494/3 827 (39) |
|  | | *5-10 years* | | 1 114/3 827 (29) |
|  | | *>10 years* | | 1 219/3 827 (32) |
| **First operator's status** | | | | |
|  | | *Nurse* | | 3 831/4 214 (91) |
|  | | *Other* | | 383/4 214 (9) |
| **Estimated number of catheters placed** | | | | |
|  | | *< 100* | | 410/4 205 (10) |
|  | | *100-800* | | 1 000/4 205 (24) |
|  | | *> 800* | | 2 795/4 205 (66) |
| **Other operator required** | | | | 187/4 216 (4) |
| **Second operator's sex (female)** | | | | 133/187 (71) |
| **Second operator's years of experience** | | | | 9 ± 8 |
| **Successfull operator different from the first** | | | | 187/4 216 (4) |
| **Successfull operator's profession** | | | | |
|  | | *Student* | | 5/187 (3) |
|  | | *Nurse* | | 179/187 (96) |
|  | | *Senior doctor* | | 3/187 (2) |
| **Estimated number of catheters placed by successful operator** | | | | |
|  | | *<100* | | 6/186 (3) |
|  | | *100-800* | | 35/186 (19) |
|  | | *>800* | | 145/186 (78) |

Data are n/N (%) or Mean ±SD. ‡ Triage scores: score 1 = most urgent score, 5 = least urgent.

# **Appendix 6. Missing data patterns and handling.**

**Missing data per centre**

Missing data were described for each covariate and across centres prior to multiple imputation. Among the 3 910 PIVCs with available outcome data, 2 619 (67·0%) had complete covariate information and were included in the complete-case analysis, corresponding to 33·0% of observations excluded due to missing covariate data.

The proportion of complete cases varied across centres, reflecting heterogeneity in data completeness at the centre level (Table 1). This variability supported the use of multiple imputation under a missing-at-random (MI-MAR) assumption, including centre as a predictor in the imputation model.

Missingness was restricted to covariates; no imputation was performed for the primary outcome.

**Table 1. Missing data per centre.**

| **Centre** | **N total** | **N complete-case** | **% complete-case** | **% with any missing covariate** |
| --- | --- | --- | --- | --- |
| 508 | 99 | 0 | 0 | 100 |
| 521 | 54 | 0 | 0 | 100 |
| 524 | 13 | 0 | 0 | 100 |
| 533 | 19 | 0 | 0 | 100 |
| 548 | 76 | 0 | 0 | 100 |
| 556 | 139 | 0 | 0 | 100 |
| 573 | 4 | 0 | 0 | 100 |
| 578 | 13 | 0 | 0 | 100 |
| 587 | 41 | 0 | 0 | 100 |
| 558 | 10 | 1 | 10 | 90 |
| 572 | 10 | 3 | 30 | 70 |
| 537 | 55 | 20 | 36 | 64 |
| 531 | 60 | 22 | 37 | 63 |
| 567 | 22 | 9 | 41 | 59 |
| 575 | 19 | 8 | 42 | 58 |
| 514 | 42 | 18 | 43 | 57 |
| 581 | 14 | 6 | 43 | 57 |
| 510 | 52 | 24 | 46 | 54 |
| 593 | 32 | 15 | 47 | 53 |
| 517 | 48 | 23 | 48 | 52 |
| 566 | 56 | 29 | 52 | 48 |
| 543 | 42 | 24 | 57 | 43 |
| 538 | 5 | 3 | 60 | 40 |
| 516 | 68 | 41 | 60 | 40 |
| 530 | 109 | 66 | 61 | 39 |
| 504 | 36 | 22 | 61 | 39 |
| 568 | 32 | 20 | 63 | 38 |
| 555 | 46 | 29 | 63 | 37 |
| 582 | 61 | 41 | 67 | 33 |
| 520 | 46 | 31 | 67 | 33 |
| 539 | 43 | 29 | 67 | 33 |
| 523 | 44 | 30 | 68 | 32 |
| 557 | 87 | 60 | 69 | 31 |
| 552 | 86 | 60 | 70 | 30 |
| 509 | 50 | 35 | 70 | 30 |
| 577 | 57 | 40 | 70 | 30 |
| 595 | 35 | 25 | 71 | 29 |
| 569 | 88 | 63 | 72 | 28 |
| 590 | 102 | 75 | 74 | 26 |
| 503 | 61 | 45 | 74 | 26 |
| 550 | 66 | 49 | 74 | 26 |
| 527 | 29 | 22 | 76 | 24 |
| 542 | 119 | 93 | 78 | 22 |
| 565 | 28 | 22 | 79 | 21 |
| 544 | 49 | 39 | 80 | 20 |
| 541 | 104 | 83 | 80 | 20 |
| 586 | 43 | 35 | 81 | 19 |
| 571 | 50 | 41 | 82 | 18 |
| 532 | 51 | 42 | 82 | 18 |
| 526 | 41 | 34 | 83 | 17 |
| 534 | 30 | 25 | 83 | 17 |
| 545 | 6 | 5 | 83 | 17 |
| 562 | 55 | 46 | 84 | 16 |
| 513 | 108 | 91 | 84 | 16 |
| 588 | 34 | 29 | 85 | 15 |
| 561 | 85 | 73 | 86 | 14 |
| 584 | 66 | 57 | 86 | 14 |
| 576 | 25 | 22 | 88 | 12 |
| 551 | 42 | 37 | 88 | 12 |
| 553 | 69 | 61 | 88 | 12 |
| 506 | 44 | 39 | 89 | 11 |
| 585 | 20 | 18 | 90 | 10 |
| 512 | 55 | 50 | 91 | 9 |
| 560 | 11 | 10 | 91 | 9 |
| 507 | 24 | 22 | 92 | 8 |
| 525 | 62 | 57 | 92 | 8 |
| 536 | 50 | 47 | 94 | 6 |
| 589 | 103 | 98 | 95 | 5 |
| 570 | 23 | 22 | 96 | 4 |
| 549 | 54 | 52 | 96 | 4 |
| 511 | 163 | 158 | 97 | 3 |
| 580 | 56 | 55 | 98 | 2 |
| 515 | 11 | 11 | 100 | 0 |
| 535 | 14 | 14 | 100 | 0 |
| 546 | 15 | 15 | 100 | 0 |
| 547 | 8 | 8 | 100 | 0 |
| 554 | 7 | 7 | 100 | 0 |
| 564 | 55 | 55 | 100 | 0 |
| 583 | 58 | 58 | 100 | 0 |

Complete-case defined as no missing values in prespecified covariates included in the multivariable model.

**Primary outcome missingness**

Among 4 216 PIVCs collected, 307 (7·3%) were excluded from the primary analysis due to missing primary outcome data. Baseline characteristics were broadly similar between included and excluded cases (Table 2). The distribution of age, body mass index, comorbidities, triage acuity, and inserter experience showed no major imbalances. Some variation was observed in selected centre-level and staffing variables; however, no consistent pattern suggested systematic exclusion of a specific clinical subgroup. Overall, these findings suggest a limited risk of substantial selection bias related to missing primary outcome data.

**Table 2. Comparison of included PIVCs and those excluded in the main analysis due to missing primary outcome data.**

|  |  | **Excluded** | **Included** | **Overall** |
| --- | --- | --- | --- | --- |
|  |  | N = 307 | N = 3 909 | N = 4 216 |
| **PATIENT CHARACTERISTICS** | |  |  |  |
| **Age (yrs)** |  |  |  |  |
|  | *<60* | 153 (50) | 1 732 (44) | 1 885 (45) |
|  | *60-75* | 62 (20) | 963 (25) | 1 025 (24) |
|  | *>75* | 90 (30) | 1 214 (31) | 1 304 (31) |
| **Body mass index (Kg/m2)** | |  |  |  |
|  | *Underweight* | 18 (7) | 186 (5) | 204 (5) |
|  | *Normal weight* | 115 (46) | 1 552 (44) | 1 667 (44) |
|  | *Overweight* | 69 (28) | 1 084 (30) | 1 153 (30) |
|  | *Obesity* | 47 (19) | 740 (21) | 787 (21) |
| **Non cooperative patient** | | 9 (3) | 139 (4) | 148 (4) |
| **Drug abuse** |  | 4 (1) | 76 (2) | 80 (2) |
| **Chemotherapy** | | 19 (6) | 238 (6) | 257 (6) |
| **Haemodialysis** | | 2 (1) | 37 (1) | 39 (1) |
| **DIVA history** |  | 41 (14) | 516 (14) | 557 (14) |
| **INSERTION CHARACTERISTICS** | |  |  |  |
| **Triage score (3 levels)** | |  |  |  |
|  | *N1 – High urgency* | 52 (20) | 682 (20) | 734 (20) |
|  | *N2 – Moderate urgency* | 98 (38) | 1 067 (32) | 1 165 (32) |
|  | *N3 – Low or non‑urgent* | 111 (43) | 1 597 (48) | 1 708 (47) |
| **Time of insertion** | |  |  |  |
|  | *Day shift* | 193 (68) | 2 811 (72) | 3 004 (72) |
|  | *Night shift* | 91 (32) | 1 067 (28) | 1 158 (28) |
| **INSERTER CHARACTERISTICS** | |  |  |  |
| **First operator's years of experience** | |  |  |  |
|  | *<5 years* | 125 (44) | 1 369 (39) | 1 494 (39) |
|  | *5-10 years* | 77 (27) | 1 037 (29) | 1 114 (29) |
|  | *>10 years* | 80 (28) | 1 139 (32) | 1 219 (32) |
| **CENTRE CHARACTERISTICS** | |  |  |  |
| **Hospital status** | |  |  |  |
|  | *University hospital* | 120 (39) | 1 650 (42) | 1 770 (42) |
|  | *Other types of hospital* | 187 (61) | 2 259 (58) | 2 446 (58) |
| **Existence of a catheter insertion protocol** | | 238 (78) | 2 772 (71) | 3 010 (71) |
| **Existence of a catheter maintenance protocol** | | 253 (82) | 2 708 (69) | 2 961 (70) |
| **Dedicated vascular access team** | | 90 (29) | 869 (22) | 959 (23) |
| **STAFFING AND ACTIVITY LEVELS** | |  |  |  |
| **Annual number of ED visits** | |  |  |  |
|  | *<15 000* | 11 (4) | 124 (3) | 135 (3) |
|  | *15 000–50 000* | 178 (58) | 2 091 (53) | 2 269 (54) |
|  | *>50 000* | 118 (38) | 1 694 (43) | 1 812 (43) |
| **Nurse-led vascular access anticipation protocol** | | 81 (26) | 612 (16) | 693 (16) |
| **Catheter placement upon ED arrival** | | 255 (83) | 2 817 (72) | 3 072 (73) |
| **Nurse-to-patient daily ratio** | |  |  |  |
|  | *Low* | 98 (32) | 1 367 (35) | 1 465 (35) |
|  | *Moderate* | 153 (50) | 1 256 (32) | 1 409 (33) |
|  | *High* | 56 (18) | 1 286 (33) | 1 342 (32) |
| **Prescriber-to-nurse ratio (day shift)** | |  |  |  |
|  | *Low* | 70 (23) | 1 531 (39) | 1 601 (38) |
|  | *Moderate* | 108 (35) | 1 114 (28) | 1 222 (29) |
|  | *High* | 129 (42) | 1 264 (32) | 1 393 (33) |
| **Prescriber-to-nurse ratio (night shift)** | |  |  |  |
|  | *Low* | 145 (47) | 1 493 (38) | 1 638 (39) |
|  | *Moderate* | 106 (35) | 1 615 (41) | 1 721 (41) |
|  | *High* | 56 (18) | 788 (20) | 844 (20) |

Baseline patient, insertion, inserter, and centre characteristics are presented according to availability of the primary outcome. Values are n (%). Included PIVCs correspond to those retained in the primary analysis; excluded PIVCs correspond to cases with missing primary outcome data. Comparisons are descriptive and intended to assess the potential for selection bias. No formal hypothesis testing was performed.

# **Appendix 7. Multivariable sensitivity analyses for factors associated with non-indicated catheter insertion.**

|  |  | **Used catheters** | **Non-indicated catheters** | **Complete-case analysis** | **Alternative centre specification** | **MNAR sensitivity analysis** | | **Alternative outcome definition** | **High-risk scenarios excluded** |
| --- | --- | --- | --- | --- | --- | --- | --- | --- | --- |
|  | | Complete-case  N = 1 722 | Complete-case  N = 897 | Complete-case  Mixed-effects logistic regression | Complete-case  Fixed-effects logistic regression | Best-case scenario | Worst-case scenario | Strict necessity (use within 24 h only) | Restricted population |
| **PATIENT CHARACTERISTICS** | |  |  |  |  |  |  |  |  |
| **Age (yrs)** |  |  |  |  |  |  |  |  |  |
|  | *<60* | 722 (42) | 425 (47) | Reference | Reference | Reference | Reference | Reference | Reference |
|  | *60-75* | 433 (25) | 215 (24) | 0·89 (0·72 - 1·10) | 0·90 (0·72 - 1·12) | 0·84 (0·69 - 1·04) | 0·84 (0·68 - 1·03) | 0·76 (0·22 - 2·68) | 1·17 (0·16 - 8·50) |
|  | *>75* | 567 (33) | 257 (29) | **0·77 (0·63 - 0·95)** | **0·78 (0·63 - 0·96)** | **0·73 (0·60 - 0·88)** | **0·73 (0·60 - 0·89)** | 1·13 (0·39 - 3·23) | 1·26 (0·23 - 6·82) |
| **BMI (Kg/m2)** | |  |  |  |  |  |  |  |  |
|  | *Underweight* | 91 (5) | 43 (5) | 1·01 (0·67 - 1·51) | 1·04 (0·69 - 1·56) | 1·26 (0·97 - 1·64) | 1·00 (0·68 - 1·49) | 0·48 (0·05 - 5·05) | 0·03 (0·00 - 63105·05) |
|  | *Normal weight* | 739 (43) | 393 (44) | Reference | Reference | Reference | Reference | Reference | Reference |
|  | *Overweight* | 546 (32) | 259 (29) | 0·92 (0·75 - 1·12) | 0·93 (0·75 - 1·14) | 0·91 (0·74 - 1·11) | 0·91 (0·74 - 1·11) | 0·41 (0·12 - 1·43) | 0·99 (0·17 - 5·69) |
|  | *Obesity* | 346 (20) | 202 (23) | 1·12 (0·89 - 1·41) | 1·12 (0·89 - 1·41) | 1·13 (0·90 - 1·42) | 1·22 (0·99 - 1·49) | 1·78 (0·57 - 5·53) | 0·95 (0·20 - 4·49) |
| **Non cooperative patient** | | 54 (3) | 32 (4) | 0·77 (0·48 - 1·23) | 0·72 (0·45 - 1·18) | 0·80 (0·55 - 1·18) | 0·82 (0·52 - 1·28) | 0·21 (0·03 - 1·41) | 6·61 (0·30 - 144·03) † |
| **Drug abuse** |  | 30 (2) | 11 (1) | 0·61 (0·29 - 1·28) | 0·64 (0·29 - 1·34) | 0·72 (0·38 - 1·33) | 0·76 (0·40 - 1·44) | 4·54 (0·33 - 62·64) | 12·09 (0·07 - 2149·02) † |
| **Chemotherapy** | | 126 (7) | 46 (5) | 0·70 (0·49 - 1·01) | 0·69 (0·48 - 1·00) | 0·73 (0·52 - 1·04) | 0·74 (0·52 - 1·05) | 0·57 (0·05 - 6·06) | 0·68 (0·03 - 16·63) |
| **Haemodialysis** | | 16 (1) | 7 (1) | 1·11 (0·44 - 2·82) | 1·14 (0·42 - 2·78) | 1·04 (0·42 - 2·60) | 1·06 (0·42 - 2·65) | Not estimable † | 132125628·29 (0·00 - ∞) |
| **DIVA history** |  | 251 (15) | 114 (13) | 0·82 (0·64 - 1·06) | 0·83 (0·64 - 1·07) | 0·82 (0·64 - 1·05) | 0·81 (0·63 - 1·04) | 0·66 (0·19 - 2·28) | 0·53 (0·07 - 4·29) |
| **INSERTION CHARACTERISTICS** | |  |  |  |  |  |  |  |  |
| **Triage score (3 levels)** | |  |  |  |  |  |  |  |  |
|  | *N1 – High urgency* | 418 (24) | 120 (13) | **0·59 (0·45 - 0·77)** | **0·57 (0·43 - 0·76)** | **0·59 (0·46 - 0·76)** | **0·59 (0·46 - 0·76)** | 0·73 (0·18 - 2·94) | 1·02 (0·12 - 8·45) |
|  | *N2 – Moderate urgency* | 567 (33) | 263 (29) | Reference | Reference | Reference | Reference | Reference | Reference |
|  | *N3 – Low or non‑urgent* | 737 (43) | 514 (57) | **1·49 (1·22 - 1·83)** | **1·44 (1·16 - 1·79)** | **1·47 (1·21 - 1·78)** | **1·46 (1·21 - 1·78)** | 2·15 (0·73 - 6·32) | 0·28 (0·04 - 1·72) |
| **Time of insertion** | |  |  |  |  |  |  |  |  |
|  | *Day shift* | 1 246 (72) | 662 (74) | Reference | Reference | Reference | Reference | Reference | Reference |
|  | *Night shift* | 476 (28) | 235 (26) | 0·94 (0·77 - 1·14) | 0·94 (0·77 - 1·15) | 0·93 (0·77 - 1·12) | 0·93 (0·78 - 1·12) | 0·39 (0·12 - 1·29) | 4·60 (0·89 - 23·69) |
| **INSERTER CHARACTERISTICS** | |  |  |  |  |  |  |  |  |
| **First operator's years of experience** | |  |  |  |  |  |  |  |  |
|  | *<5 years* | 677 (39) | 338 (38) | 0·98 (0·79 - 1·22) | 1·01 (0·81 - 1·27) | 1·02 (0·83 - 1·26) | 1·02 (0·83 - 1·26) | 0·50 (0·15 - 1·71) | 18·59 (1·64 - 210·17) |
|  | *5-10 years* | 501 (29) | 263 (29) | Reference | Reference | Reference | Reference | Reference | Reference |
|  | *>10 years* | 544 (32) | 296 (33) | 1·02 (0·82 - 1·29) | 1·07 (0·84 - 1·35) | 1·09 (0·87 - 1·35) | 1·09 (0·88 - 1·35) | 0·23 (0·05 - 0·96) | 37·91 (3·47 - 414·48) † |
| **CENTRE CHARACTERISTICS** | |  |  |  |  |  |  |  |  |
| **Hospital status** | |  |  |  |  |  |  |  |  |
|  | *University hospital* | 748 (43) | 372 (42) | Reference | Reference | Reference | Reference | Reference | Reference |
|  | *Other types of hospital* | 974 (57) | 525 (59) | 0·99 (0·71 - 1·38) | 6·45 (0·17 - 281·46) | 1·02 (0·73 - 1·42) | 1·02 (0·73 - 1·43) | 3·94 (0·20 - 79·10) | 0·19 (0·02 - 2·07) |
| **Existence of a catheter insertion protocol** | | 1 151 (66·8) | 573 (64) | 1·07 (0·47 - 2·47) | 0·04 (0·00 - 48·15) | 1·02 (0·43 - 2·40) | 1·03 (0·44 - 2·42) | 0·12 (0·00 - 217532·89) † | 75·48 (0·00 - 5062437218·36) † |
| **Existence of a catheter maintenance protocol** | | 1 146 (67) | 555 (62) | 0·84 (0·36 - 1·95) | 2·98 (0·14 - 69·75) | 0·92 (0·39 - 2·19) | 0·91 (0·39 - 2·15) | 2·81 (0·00 - 4779972·18) † | 0·02 (0·00 - 1370652·36) † |
| **Dedicated vascular access team** | | 447 (26) | 160 (18) | **0·49 (0·32 - 0·73)** | 1287·87 (0·03 - 100342619·26) † | **0·47 (0·31 - 0·72)** | **0·47 (0·31 - 0·72)** | 0·28 (0·01 - 5·98) | 7·84 (0·64 - 95·60) |
| **STAFFING AND ACTIVITY LEVELS** | |  |  |  |  |  |  |  |  |
| **Annual number of ED visits** | |  |  |  |  |  |  |  |  |
|  | *<15 000* | 56 (3) | 31 (4) | 0·74 (0·38 - 1·45) | 2·39 (0·75 - 7·88) | 0·70 (0·35 - 1·37) | 0·69 (0·35 - 1·37) | 0·92 (0·01 - 72·90) | 0·15 (0·00 - 16·01) |
|  | *15 000–50 000* | 905 (53) | 480 (54) | Reference | Reference | Reference | Reference | Reference | Reference |
|  | *>50 000* | 761 (44) | 386 (43) | 1·09 (0·77 - 1·55) | 0·08 (0·00 - 5·20) | 1·10 (0·77 - 1·58) | 1·10 (0·77 - 1·57) | 0·09 (0·01 - 1·72) | 127·89 (6·77 - 2415·38) † |
| **Nurse-led vascular access anticipation protocol** | | 303 (18) | 115 (13) | 0·68 (0·44 - 1·05) | 2·74 (0·53 - 14·49) | 0·73 (0·47 - 1·12) | 0·72 (0·47 - 1·12) | 0·62 (0·02 - 17·45) | 0·26 (0·03 - 2·18) |
| **Catheter placement upon ED arrival** | | 1 311 (76) | 697 (78) | 1·13 (0·77 - 1·66) | 0·01 (0·00 - 24·26) | 1·03 (0·70 - 1·52) | 1·03 (0·70 - 1·52) | 0·68 (0·03 - 14·27) | 52·16 (0·66 - 4118·95) † |
| **Nurse-to-patient daily ratio** | |  |  |  |  |  |  |  |  |
|  | *Low* | 667 (39) | 367 (41) | 1·49 (1·01 - 2·20) | 0·70 (0·16 - 2·97) | 1·52 (1·02 - 2·25) | 1·51 (1·02 - 2·23) | 14·00 (0·44 - 449·64) | 0·01 (0·00 - 0·28) |
|  | *Moderate* | 571 (33) | 278 (31) | Reference | Reference | Reference | Reference | Reference | Reference |
|  | *High* | 484 (28) | 252 (28) | 1·16 (0·78 - 1·72) | 0·08 (0·00 - 5·47) | 1·20 (0·80 - 1·80) | 1·20 (0·81 - 1·80) | 0·59 (0·03 - 12·92) | 5·39 (0·21 - 138·85) † |
| **Prescriber-to-nurse ratio (day shift)** | |  |  |  |  |  |  |  |  |
|  | *Low* | 706 (41) | 364 (41) | 1·19 (0·77 - 1·82) | 241·37 (0·18 - 444601·64) † | 1·21 (0·79 - 1·87) | 1·22 (0·79 - 1·87) | 68·26 (0·82 - 5711·67) † | 0·01 (0·00 - 0·92) |
|  | *Moderate* | 427 (25) | 215 (24) | Reference | Reference | Reference | Reference | Reference | Reference |
|  | *High* | 589 (34) | 318 (36) | 1·05 (0·70 - 1·59) | 9226·14 (0·04 - 4156799540·79) | 1·11 (0·73 - 1·69) | 1·11 (0·73 - 1·68) | 5·60 (0·06 - 487·00) † | 0·20 (0·00 - 14·28) |
| **Prescriber-to-nurse ratio (night shift)** | |  |  |  |  |  |  |  |  |
|  | *Low* | 697 (41) | 338 (38) | 0·90 (0·61 - 1·33) | 693·79 (0·10 - 7257207·59) † | 0·89 (0·60 - 1·32) | 0·89 (0·60 - 1·33) | 15·11 (0·36 - 631·52) † | 0·02 (0·00 - 0·53) |
|  | *Moderate* | 587 (34) | 356 (40) | Reference | Reference | Reference | Reference | Reference | Reference |
|  | *High* | 438 (25) | 203 (23) | 0·92 (0·61 - 1·38) | 13·84 (1·29 - 180·73) | 0·89 (0·59 - 1·35) | 0·89 (0·59 - 1·34) | 4·38 (0·13 - 149·68) † | 0·48 (0·02 - 9·38) |

Data are n (%). Bold values are presented for ease of reading and do not imply statistical significance.

† Estimates from fixed-effects centre models were unstable due to sparse data and quasi-separation within centres; results should be interpreted with caution.

aOR: adjusted odds ratio; CI: confidence interval; ED: Emergency Department; MNAR: Missing Not At Random

# **Appendix 8. Centre-level variability in non-indicated PIVC placement (Panel A) and enrolled patients (Panel B)**

| **Center** | **Non-indicated catheters** | |
| --- | --- | --- |
|  | **n/N (%)** | **95% CI** |
| 503 | 13/61 (21%) | 13%–33% |
| 504 | 2/36 (6%) | 2%–18% |
| 506 | 15/44 (34%) | 22%–49% |
| 507 | 4/24 (17%) | 7%–36% |
| 508 | 32/99 (32%) | 24%–42% |
| 509 | 25/50 (50%) | 37%–63% |
| 510 | 18/52 (35%) | 23%–48% |
| 511 | 38/162 (23%) | 18%–31% |
| 512 | 26/55 (47%) | 35%–60% |
| 513 | 38/103 (37%) | 28%–47% |
| 514 | 15/36 (42%) | 27%–58% |
| 515 | 1/10 (10%) | 2%–40% |
| 516 | 14/68 (21%) | 13%–32% |
| 517 | 17/48 (35%) | 23%–50% |
| 518 | NA | NA |
| 520 | 19/46 (41%) | 28%–56% |
| 521 | 2/45 (4%) | 1%–15% |
| 522 | NA | NA |
| 523 | 12/44 (27%) | 16%–42% |
| 524 | 1/13 (8%) | 1%–33% |
| 525 | 25/62 (40%) | 29%–53% |
| 526 | 0/41 (0%) | 0%–9% |
| 527 | 16/29 (55%) | 38%–72% |
| 530 | 48/109 (44%) | 35%–53% |
| 531 | 25/60 (42%) | 30%–54% |
| 532 | 20/51 (39%) | 27%–53% |
| 533 | 9/19 (47%) | 27%–68% |
| 534 | 3/25 (12%) | 4%–30% |
| 535 | 4/14 (29%) | 12%–55% |
| 536 | 20/48 (42%) | 29%–56% |
| 537 | 22/55 (40%) | 28%–53% |
| 538 | 0/5 (0%) | 0%–43% |
| 539 | 18/43 (42%) | 28%–57% |
| 541 | 34/104 (33%) | 24%–42% |
| 542 | 34/119 (29%) | 21%–37% |
| 543 | 15/42 (36%) | 23%–51% |
| 544 | 0/49 (0%) | 0%–7% |
| 545 | 0/6 (0%) | 0%–39% |
| 546 | 0/15 (0%) | 0%–20% |
| 547 | 2/8 (25%) | 7%–59% |
| 548 | 21/76 (28%) | 19%–39% |
| 549 | 27/54 (50%) | 37%–63% |
| 550 | 4/50 (8%) | 3%–19% |
| 551 | 9/42 (21%) | 12%–36% |
| 552 | 33/86 (38%) | 29%–49% |
| 553 | 21/66 (32%) | 22%–44% |
| 554 | 3/7 (43%) | 16%–75% |
| 555 | 17/46 (37%) | 25%–51% |
| 556 | 57/138 (41%) | 33%–50% |
| 557 | 41/86 (48%) | 37%–58% |
| 558 | 4/10 (40%) | 17%–69% |
| 560 | 0/11 (0%) | 0%–26% |
| 561 | 20/85 (24%) | 16%–34% |
| 562 | 26/54 (48%) | 35%–61% |
| 564 | 22/55 (40%) | 28%–53% |
| 565 | 11/27 (41%) | 25%–59% |
| 566 | 16/56 (29%) | 18%–41% |
| 567 | 6/22 (27%) | 13%–48% |
| 568 | 22/32 (69%) | 51%–82% |
| 569 | 29/88 (33%) | 24%–43% |
| 570 | 5/23 (22%) | 10%–42% |
| 571 | 18/50 (36%) | 24%–50% |
| 572 | 1/10 (10%) | 2%–40% |
| 573 | 1/4 (25%) | 5%–70% |
| 575 | 4/15 (27%) | 11%–52% |
| 576 | 13/24 (54%) | 35%–72% |
| 577 | 0/35 (0%) | 0%–10% |
| 578 | 2/13 (15%) | 4%–42% |
| 580 | 16/56 (29%) | 18%–41% |
| 581 | 4/14 (29%) | 12%–55% |
| 582 | 22/61 (36%) | 25%–49% |
| 583 | 33/57 (58%) | 45%–70% |
| 584 | 40/66 (61%) | 49%–71% |
| 585 | 10/20 (50%) | 30%–70% |
| 586 | 11/43 (26%) | 15%–40% |
| 587 | 19/41 (46%) | 32%–61% |
| 588 | 10/32 (31%) | 18%–49% |
| 589 | 33/103 (32%) | 24%–42% |
| 590 | 49/102 (48%) | 39%–58% |
| 593 | 17/32 (53%) | 36%–69% |
| 595 | 14/33 (42%) | 27%–59% |

**Panel A**

**Panel B**
