# Supplementary material for: Peripheral intravenous catheter use in French emergency departments (CathIRU study): a multicentre cross-sectional study of non-indicated insertion rate and practice patterns
Source: Lancet Reg Health Eur. 2026 Apr 10;65:101674. doi: 10.1016/j.lanepe.2026.101674 (PMC13091371; doi:10.1016/j.lanepe.2026.101674)
Supplement: List of Collaborators [file mmc2.docx]

**LIST OF COLLABORATORS**

| **Last name** | **First name** | **Degree** | **Affiliation** | **Mail address** |
| --- | --- | --- | --- | --- |
| RAYNAUD | Camille | MD | CHU de Poitiers, Services des urgences Adultes SAS 86, Poitiers, France | camilleraynaud@hotmail.com |
| MORVAN | Maelle | MD | CHU de Poitiers, Services des urgences Adultes SAS 86, Poitiers, France | maelle.morvan@wanadoo.fr |
| COUVREUR | Raphael | MD | CHU de Poitiers, Services des urgences Adultes SAS 86, Poitiers, France | raphael.couvreur2@gmail.com |
| DEMARQUET | Marine | MD, MSc | CH Bourg-en-Bresse, Service d'accueil des urgences, Bourg-en-Bresse, France | marine.demarquet@gmail.com |
| LIMOUSIN | Johan | NP | CH Départemental Vendée, Service d'accueil des urgences, La Roche sur Yon, France | johan.limousin@ght85.fr |
| ROCARD | Fabien | RN | CH Côte de Lumière, Service d'accueil des urgences, Les Sables d'Olonne, France | fabien.rocard@ght85.fr |
| CARROUEE | Ludivine | RN | CH Départemental Vendée, Service d'accueil des urgences, Luçon, France | ludivine.carrouee@ght85.fr |
| VOISIN | Vanessa | RN | CH Loire Vendée Océan, Service d’accueil et de traitement des urgences, Challans, France | vanessa.voisin@ght85.fr |
| NAULIN-LERAY | Claire | NP | CH Départemental Vendée, Service d'accueil des urgences, Montaigu, France | claire.naulin@ght85.fr |
| GRANGER | Fanny | NP | CH Pierre Oudot, Service des urgences, Bourgoin-Jallieu, France | fgranger@ghnd.fr |
| ROCHARD | Clément | NP | CH Angoulême, Service des urgences, Angoulême, France | clement.rochard@ch-angouleme.fr |
| SUSONG | Ombeline | MD | GHT ATLANTIQUE 17 - Service des urgences, La Rochelle,France | ombeline.susong@gmail.com |
| CHENU | Mathilde | MD | GHT ATLANTIQUE 17 - Service des urgences, Rochefort, France | mathilde9.chenu@gmail.com |
| ADAM | Romain | MD | CHU de Besançon, Service des urgences Adultes, Besançon, France | radam@chu-besancon.fr |
| JOURDAIN | Matthieu | MD | CH Valenciennes, service des urgences SMUR, Valenciennes, France | jourdain-m@ch-valenciennes.fr |
| RUIZ | Guillaume | RN | CHU de Nîmes, Service des urgences, Nîmes, France | ruiz.guillaume@hotmail.fr |
| DUBUCS | Xavier | MD, MSc | CHU de Toulouse, Service des urgences, Toulouse, France | xavier.dubucs@gmail.com |
| WEMMERT | Charlotte | MD | CH Victor Dupouy, Equipe Mobile d'Antibiothérapie, Argenteuil, France | charlotte.wemmert@ch-argenteuil.fr |
| MULLER | Delphine | RN | CH Gourdon, Service des urgences, Gourdon, France | delphine.muller@ch-gourdon.fr |
| GOSSELIN | Séverine | MD | CHU de Dijon, Département Universitaire de Médecine d'Urgence, Dijon, France | severine.gosselin@chu-dijon.fr |
| PEREIRA | Xavier | MD | CH Côte Basque, Services des urgences SAMU-SMUR, Bayonne, France | xpereira@ch-cotebasque.fr |
| LIBE | Céline | RN | Clinique générale de Marignane, Services des urgences, Marignane, France | cadre.urgences.marignane@almaviva-sante.com |
| PAPIN | Mathilde | MD | CHU de Nantes, Service des urgences-SAMU, Nantes, France | mathilde.papin@chu-nantes.fr |
| GERLIER | Camille | MD | Hopital Paris Saint Joseph, Service des Urgences, Paris, France | cgerlier@ghpsj.fr |
| DUJARDIN | Coraline | RN | CHCP, service des urgences, Comminges, France | coraline.dujardin@ch-saintgaudens.fr |
| NOEL | Florent | MD | AP-HP.Sorbonne Université, Hôpital Saint-Antoine, Service d'Accueil des Urgences, Paris, France. | florent.noel@aphp.fr |
| BURGGRAFF | Eric | MD | Hôpital Tenon, Services des Urgences, Paris, France | eric.burggraff@aphp.fr |
| ATTIA | Delphine | MD | AP-HP. Université Paris Saclay, Hôpital Antoine Béclère, Service d'Accueil des Urgences, Clamart, France | delphine.attia@aphp.fr |
| PEYRONY | Olivier | MD | Hôpital Saint Louis, Services des Urgences, Paris, France | o.peyrony@hotmail.fr |
| HAMED | Rym | MD | CH Louis Pasteur, Chartres, Le Coudray, France | rhamed@ch-chartres.fr |
| GARROUSTE | Vincent | MD | CHU d'Orléans, Services des urgences Adultes, Orléans, France | vincent.garrouste@chu-orleans.fr |
| MANDIN | Rébecca | MD | CH Ardèche Meridionale, Pharmacie, Aubenas, France | rebecca.levy@ch-ardeche-meridionale.fr |
| FUTIN | Romain | MD | Groupe Hospitalier Île de France Sud - Centre Hospitalier Sud Francilien, Corbeil-Essonnes, France | romain.futin@gmail.com |
| ANDRIANJAFY | Hery | MD | Groupe Hospitalier IIe de France sud - CH Arpajon, Service des urgences, Arpajon, France | handrianjafy@ch-arpajon.fr |
| DEBBABI | Haithem | MD | CH Brioude, service des Urgences, France | [hdebbabi@ch-brioude.fr](mailto:hdebbabi@ch-brioude.fr) |
| FREMERY | Alexis | MD | CHU Guyane, Service des Urgences, Cayenne, Guyane Française, France | alexis.fremery@ch-cayenne.fr |
| DUPONT | Pierre-yves | MD | CH William Morey, Service d'accueil des urgences, Chalon sur Saône, France | pierre-yves.dupont@ch-chalon71.fr |
| ABBAL | Frédéric | MD | CH Lavaur, Service des urgences - SMUR, Lavaur, France | f.abbal@ch-lavaur.fr |
| KWAKYE AGYEMANG | Angelo giordano | MD | CH Émile Roux Le-Puy-en-Velay, Service d'accueil des urgences, Le-Puy-en-Velay, France | [ag.kwakyeagyemang@ch-lepuy.fr](mailto:ag.kwakyeagyemang@ch-lepuy.fr) |
| NEGRELLO | Florian | MD | CHU de Martinique, service des urgences, Fort de France, France | florian.negrello@chu-martinique.fr |
| LESAGE | Patrick | MD | CHMS - Service des Urgences, Chambéry, France | patrick.lesage@ch-metropole-savoie.fr |
| SEBBANE | Mustapha | MD,PHD | CHU de Montpellier, Urgences/SMUR, Montpellier, France | m-sebbane@chu-montpellier.fr |
| THIRIEZ | Sylvain | MD | CH de Roubaix, Service des urgences, Roubaix, France | sylvain.thiriez@ch-roubaix.fr |
| GALINSKI | Christelle | RN | CH de Mortagne, Service des Urgences et SMUR, Mortagne, France | chriseel2@wanadoo.fr |
| GONFRÈRE | Gwendoline | MD | CH de Rodez, service des urgences, Rodez, France | g.gonfrere@ch-rodez.fr |
| LEVY | Delphine | MD | Hôpital Saint Joseph, Service des Urgences, Marseille, France | dlevy@hopital-saint-joseph.fr |
| RENAULT FRADIN | Maï | RN | CH Pau, Direction des Soins, Pau, France | mai.fradin@ch-pau.fr |
| BONNAURE-SORBIER | Alice | MD | CH Saintes, Service des urgences et SMUR, Saintes, France | alice.bonnaure-sorbier@gh-saintesangely.fr |
| JEANNE | Sewann | MD | CH Robert Boulin, service des Urgences, Libourne, France | sewannjeanne@gmail.com |
| RIO | Gwenola | NP | CHU de Rennes, Service des Urgences Adultes, Rennes, France | Gwenola.BLOT.RIO@chu-rennes.fr |
| LARIBI | Said | MD, PhD | CHU Tours, Service des Urgences, Tours, France | s.laribi@chu-tours.fr |
| LECLERCQ-MARTIN | Aurélien | MD | CH Soissons, Service d'Accueil des Urgences, Soissons, France | aurelien.martin-kleisch@ch-soissons.fr |
| BERMENT | Lea | RN | CH Verneuil sur Avre, Service des urgences, Verneuil sur Avre, France | Lea.berment@vbr-sudeure.fr |
| SAMAIN | Julie | MD | CH Vesoul - Groupement Hospitalier de la Haute-Saone, Service des Urgences, Vesoul, France | j.samain@gh70.fr |
| HELIOS | Bertrand | NP | CHU de Reims, Service des Urgences Adultes, Reims, France | bhelios@chu-reims.fr |
| BORTZMEYER | Jessica | RN | CHU de Rouen, Service des urgences, Rouen, France | Jessica.Bortzmeyer@chu-rouen.fr |
| VIOLEAU | Mathieu | MD | CH de Niort, Services des Urgences-SAMU-SMUR, Niort, France | mathieu.violeau@ch-niort.fr |
| BOUNAUD | Nicolas | MD | CH de Carcassonne, Service des urgences, Carcasonne, France | bounaud.n@oruoccitanie.fr |
| KACED | Manon | NP | Hospices Civils de Lyon, Hôpital Edourd Herriot, Service des urgences, Lyon, France | manon.kaced@chu-lyon.fr |
| LEON | Morgane | NP, MSc | CHU Lyon Sud - Hospices civils de Lyon , Service des urgences , Lyon, France | morgane.leon@chu-lyon.fr |
| PRUDHOMME | Elise | NP | Hospices Civils de Lyon, Hôpital de la Croix-Rousse, Service des Urgences, Lyon, France | elise.prudhomme@chu-lyon.fr |
| CAMPHUIS | Antonin | MD | CH de Bethune, Service d'Accueil des Urgences - SMUR, Bethune, France | acamphuis@ch-bethune.fr |
| ROUX-BONIFACE | Daniel | MD | CHU Clermont-Ferrand, Pôle Urgences-SAMU-SMUR, Clermond-Ferrand, France | droux-boniface@chu-clermontferrand.fr |
| RESTOUILH | Virginie | NP | CHU de Strasbourg, Service des urgences adultes, Strasbourg, France | virginie.restouilh@chru-strasbourg.fr |
| BARTH | Arnaud | RN | CH de Sélestat, Structure des Urgences, Sélestat, France | arnaud.barth1@gmail.com |
| BERRARD-LORRILLERE | Olivia | MD | Polyclinique de Poitiers, Service des urgences, Poitiers, France | oberrard@hotmail.com |
| CAZES | Nicolas | MD | Hôpital d’Instruction des Armées Laveran, Service des Urgences, Marseille, France | nicolas.cazes@icloud.com |
| YVON | Julien | MD | CH Nord Ouest Tarare, Services des Urgences, Tarare, France | jyvon@hno.fr |
| HERMANN | William | NP | CH Emile Muller, GHRMSA, pôle ARUBA, SAMU, SMUR, Urgences de Mulhouse, Mulhouse, France | william.hermann@ghrmsa.fr |
| FERTAT | Margaux | MD | CH Annecy Genevois, Service des Urgences, Annecy, France | mfertat@ch-annecygenevois.fr |
| FROMONT | Isaure | MD | CH Annecy Genevois 2, Service des Urgences, Annecy, France | ifromont@ch-annecygenevois.fr |
| HENRY | Aline | RN | CH Melun, Groupe Hospitalier Sud-ile-de-France, Service des urgences, Melun, France | aline.henry@ghsif.fr |
| VILLARD | Isabelle | RN | Hopital NOVO-Site de Pontoise, Service des Urgences, Cergy-Pontoise, France | isabelle.villard@ght-novo.fr |
| TOUIHAR | Ayoub | MD | CH Auxerre, Service des urgences, Auxerre, France | ayoub.touihar@gmail.com |
| PALOMERA | Adrien | MD | CH de Marmande, Service des urgences, Marmande, France | palo7@hotmail.fr |
| VAN CAENEGEM | Pierre | MD | CH Versailles, Hôpital André Mignot, Service des Urgences, Le Chesnay, France | pvancaenegem@ght78sud.fr |
| SARTORIUS | Max- antoine | MD | CJITS Sainte Musse, Pole médecine d'urgence, La Seyne sur mer, France | max-antoine.sartorius@ch-toulon.fr |
| BAISSE | Arthur | MD | CH Albi, Service des Urgences, Albi, France | arthur.baisse@ch-albi.fr |
| JAEGER | Deborah | MD, MSc, PhD | CHRU Nancy, Service d'Urgences, Nancy, France | drdeborahjaeger@gmail.com |
| GOFFIN | Pierre | MD, MSc, PhD (cand) | Hopital Montlegia, Groupe Santé CHC, Service d'anesthesie et soins intensifs, Liège, Belgique, | goffin.pjc@gmail.com |
